# Metabolite Fingerprinting of Kersting's Groundnut [*Macrotyloma geocarpum* (Harms) Maréchal & Baudet] Seeds Using UPLC-qTOF-MS Reveals the Nutraceutical and Antioxidant Potentials of the Orphan Legume

**DOI:** 10.3389/fnut.2020.593436

**Published:** 2020-12-15

**Authors:** Armelle Tontsa Tsamo, Mustapha Mohammed, Felix Dapare Dakora

**Affiliations:** ^1^Department of Organic Chemistry, University of Yaoundé I, Yaounde, Cameroon; ^2^Department of Chemistry, Tshwane University of Technology, Pretoria, South Africa; ^3^Department of Crop Sciences, Tshwane University of Technology, Pretoria, South Africa

**Keywords:** underutilized legumes, *Macrotyloma geocarpum*, UPLC-qTOF-MS, antioxidants, phenolic compounds, flavonoids, fatty acids, sphingolipids

## Abstract

The identification and subsequent quantification of phenolic compounds in plants is the first step toward harnessing their associated nutritional and health benefits. Due to their diverse phenolic compound compositions, grain legumes are known for their high nutritional and health values. The aim of this study was to assess the inter-variations in chemical composition, phytochemical content, and antioxidant capacity of seed extracts from eight Kersting's groundnut [*Macrotyloma geocarpum* (Harms) Marechal & Baudet] landraces. The chemical profiles were evaluated using UPLC-qTOF-MS. Total phenolics and flavonoids content were determined by the Folin-Ciocalteu and aluminum chloride methods, respectively. The antioxidant capacities in the forms of DPPH and ABTS were evaluated using spectrophotometric methods. Principal component analysis was used to define similarities/differences between the landraces. Based on untargeted metabolomics analysis, 57 metabolites were identified, with phenolics, triterpenes, fatty acids, and sphingolipids being the most predominant. The results showed that the black seeded KG1 (Puffeun) had the highest total phenolic (9.44 mg GAE/g) and flavonoid (3.01 mg QE/g) contents, as well as antioxidant capacity (9.17 μg/mL and 18.44 μg/mL based on DDPH and ABTS assays, respectively). The concentrations of ferulic acid hexoside, procyanidin B2, eryodictyiol-7-rutinoside and quercetin pentoside ranged from 51.78–441.31, 1.86–18.25, 3.26–13.95 to 5.44–63.85 μg/mg, respectively. This study presents a useful report on the phytochemical characterization of Kersting's groundnuts and shows that the grains can be used as a source of nutraceuticals for human consumption.

## Introduction

Legumes have long been known for their nutritional and health benefits through the provision of proteins and mineral elements in human diets (1–3). The nutritional benefits of grain legumes may be attributed to their phytochemical compositions that often confer biological activities of interest (4, 5). For example, most legumes are known to exhibit a wide range of seed pigmentation due to differences in the relative concentrations of anthocyanins, a flavonoid with multiple benefits for plant protection and human health (6–8). Moreover, legume seeds contain a diverse range of phenolics which may be classified according to their chemical structures as flavonoids (anthocyanins, flavones, and flavanols), phenolic acids, stilbenes, and tannins (9). In the plant system, phenolic compounds act as phytoalexins, antifeedants, attractants for pollinators, growth regulators, contributors to plant pigmentation, antioxidants, and protective agents against UV light (10, 11). They are also known to modulate several pathophysiological processes in humans, including inflammation, oxidative stress, blood pressure, microbial growth, mutagenic processes, as well as the reduction in the risk of chronic metabolic and degenerative diseases (12). Phenolic compounds also contribute to the bitterness, astringency, color, flavor, odor, and oxidative stability of food products (5, 13). Aside the nutraceutical benefits of phenolic and flavonoid compounds, they are also employed during the ensuing processes that lead to nodulation in the legume-rhizobia symbiosis (14, 15).

Legume seeds have been found to exhibit higher antioxidant activity among selected crops and have significant total phenolic content (16–18). Since antioxidant capacity in various legume seeds is directly linked to their total phenolic, flavonoid, and anthocyanin contents (17), the consumption of such legume products could contribute to the management and/or prevention of several chronic and degenerative diseases, in addition to their traditional role in the prevention of protein-calorie malnutrition (2). Like other phytochemicals, fatty acids, and sphingolipids also present various health related benefits in humans and are known to play an important role in complex metabolic processes. However, whereas saturated fatty acids often pose adverse health effects, the unsaturated fatty acids have protective roles (19). Due to their wide range of functions and biological properties, sphingolipids play vital roles in human systems, including the management of diseases such as cancer, obesity, and atherosclerosis (20).

In addition to the pressure on agricultural systems to produce enough food for the growing human population, the high demand for healthy foods has also led to increased efforts to develop or bio-prospect for crop genotypes with increased phenolic concentrations (21, 22). For example, recent breeding efforts have focused on incorporating biochemical pathways involved in the biosynthesis of desired phytochemicals in cultivated crops (23). However, the identification of crop genotypes showing a wide variation in phytochemical compositions offers an opportunity for their incorporation into other species. For this reason, there is renewed interest in the search for useful biological traits such as increased levels of biologically desired phytochemicals among underutilized legumes for improved human nutrition and health (24–27).

Kersting's groundnut (KG) is an under-utilized grain legume indigenous to sub-Saharan Africa (28). Grown for its seeds at the subsistence level, KG is known to possess high nutritional, medicinal and cultural values. In some parts of Africa, water from the boiled KG seeds is used for the treatment of diarrhea (29). Given its nutritional and medicinal attributes as well as the variable seed coat pigmentation, KG could be a source of natural antioxidants for daily consumption (8). In addition to its nutritional value, KG is capable of forming root nodules when in symbiosis with rhizobia, leading to the reduction of atmospheric N_2_ to NH_3_ for the benefit of cropping systems (30–32). There is however little information on the metabolite profile of KG seeds and their bioactivities, an aspect much needed to tap the nutraceutical benefits of this underutilized legume.

The aim of this study was to identify and characterize the metabolites in the edible seeds of eight KG landraces exhibiting variable seed coat pigmentation. The antioxidant capacity of the seed extracts was estimated using two independent assays: 2,2-diphenyl-1-picrylhydrazyl (DPPH) and 2,2′-azino-bis (3-ethylbenzothiazoline-6-sulphonic acid) diammonium salt (ABTS). The differences in the chemical profiles of the test landraces was assessed by means of principal component analysis (PCA). This study reports the phytochemical composition and the antioxidant activities of KG seeds and highlights their potential utilization as nutraceuticals.

## Materials and Methods

### Chemicals and Reagents

Formic acid and all solvents used were of the LC-MS grade. Ultrapure water (resistivity of 18.2 MΩ cm^−1^ at 25°C) obtained from a Millipore water purification system was used. Standards of phenolic acids (gallic acid, caffeic acid, quinic acid, *p*-coumaric acid, ferulic acid, and sinapic acid) and flavonoids (catechin, epicatechin, rutin, naringin, gallocatechin, and eryodictyol-7-rutinoside) were obtained from Sigma Chemicals Co. (Germany). Sodium carbonate, potassium ferric cyanide, iron (II) sulfate, aluminum chloride, and hydrochloric acid were obtained from Merck (South Africa), while 2,2-azino-bis (3-ethylben-zothiazoline-6-sulfonic acid) diammonium salt (ABTS), 2,2-diphenyl-1-picrylhydrazyl (DPPH), Folin-Ciocalteu's reagent, gallic acid, 2,5,7,8-tetramethylchroman carboxylic acid (Trolox), potassium persulfate, glacial acetic acid, ascorbic acid, methanol, and ethanol were sourced from Sigma-Aldrich (St. Louis, MO, USA). All other solvents and chemicals used were of the reagent grade and purchased from Sigma-Aldrich (Germany).

### Sample Materials

The eight (8) KG landraces (Table 1, Supplementary Figure 1) used in this study were sourced from the University for Development Studies (Nyankpala, Ghana). The landraces were previously collected from communities across the Upper West region of Ghana and described according to their morphological and genetic traits (32, 33). The identification of the plants was done by examining the morphological characteristics of their flowers, leaves and seed structures. Voucher specimens (TUTMG1401-TUTMG1408), corresponding to KG1–KG8, respectively, were assigned to the seeds and deposited at the Herbarium of the Department of Crop Sciences, Faculty of Science, Tshwane University of Technology, Pretoria, South Africa.

**Table 1:** Geographic origin and characteristics of the Kersting's groundnut (KG) landraces reported in this study.

**Sample code**	**Local names**	**Seed weight^a^**	**Seed phenotypes**	**Origin**	**Geographic coordinates**	
**–**	**–**	**(g/100 seeds)**	**(color)**	**District**	**Longitude**	**Latitude**
KG1	Puffeun	11.2 ± 0.74	Black	Lawra	10.6459	−2.8827
KG2	Boli	15.1 ± 0.01	White	Wa	10.0601	−2.5099
KG3	Funsi	14.3 ± 0.38	Brown mottled	Wa East	9.9858	−1.9099
KG4	Sigiri	16.1 ± 0.64	Brown mottled	Jirapa	10.5238	−2.7034
KG5	Nakori	17.0 ± 0.38	Brown mottled	Wa	10.0601	−2.5099
KG6	Heng Milk Mottled	16.5 ± 1.04	Brown mottled	Nandom	10.8526	−2.7606
KG7	Dowie	16.5 ± 0.89	Brown mottled	Sisala West	10.5229	−2.0665
KG8	Belane Mottled	13.6 ± 0.93	Brown mottled	Nadowli	10.3669	−2.6636

a *Seed weight is expressed as the mean ± standard deviation of triplicate weights of 100 seeds. Seed photos are provided in Supplementary Figure 1*.

To obtain fresh seeds for this study, each landrace was sown in three replicate plots measuring 2.4 m × 2 m at a spacing of 60 cm between rows and 20 cm between plants. Planting was done in open field experiments in June 2014 at Nyankpala in the Northern Region of Ghana. At maturity (120 days after sowing), plants were harvested, and the pods collected. Pods were sun-dried and threshed to obtain the seeds, which were then dried to 13% moisture content prior to determination of phenolic compounds in the laboratory. For each landrace, three biological samples were analyzed separately.

### Sample Extraction

Extracts were obtained from the seeds of KG landraces harvested from the field experiments in Ghana. The seeds were ground using a coffee grinder (Cuisinart, model DCG-20N series). Approximately 30 g of ground KG were extracted thrice by maceration with 200 mL of 1% trifluoroacetic acid (TFA) in CH_3_OH/H_2_O (80:20). The mixture was then sonicated twice for 2 h and left at room temperature (20°C) for 24 h under constant agitation on a shaker. The extract was centrifuged for 15 min at 4,000 rpm and the supernatant collected. The resulting solutions were combined and evaporated to dryness under vacuum using a Buchi rotavapor R-100 (Buchi Labortechnik, Flawil, Switzerland) at 40°C and stored at 4°C prior to use within 24 h (34). One milligram of dried extracts was re-dissolved in 1 mL of CH_3_OH/H_2_O (1:1, v/v), filtered through a 0.22 μm syringe filter and transferred directly into HPLC vials (2 mL) before being analyzed by ultra-performance liquid chromatography.

To assess biological variance, 3 biological replicates for each sample were extracted and analyzed in parallel under identical conditions.

### Identification of Phenolic Compounds in Seeds of Kersting's Groundnut

#### Qualitative Analysis Using UPLC-qTOF-MS

Chromatographic analysis was performed on a Waters Acquity Ultra Performance Liquid Chromatographic system with PDA detector (USA) coupled to MS detector (Waters Corporation, H-Class Bio System, Milford, USA). Separation was achieved on an Acquity UPLC BEH C18 column (150 mm × 2.1 mm, i.d., 1.7 μm particle size, Waters) maintained at 40°C. Preliminary tests were done prior to setting of the chromatographic conditions to obtain chromatograms with better resolution and short analysis time. The mobile phase consisted of 0.1% trifluoroacetic acid (solvent A) and acetonitrile (solvent B) at a flow rate of 0.3 mL/min. The gradient elution was executed as follow: initial ratio was 90% A: 10% B, keeping for 1 min., changed to 25% A: 75% B in 10 min., to 5% A: 95% B in 5 min., keeping for 1 min and back to initial ratio in 0.5 min., with the equilibration of the system for 1.5 min. The total running time was 18 min. with an injection volume of 2.0 μL (full-loop injection). The positive and negative ion modes were examined, and the negative ion mode found to yield results with more information and higher sensitivity. Thus, the mass spectrometry was operating in a negative ion electrospray mode and nitrogen (N_2_) was used as the desolvation gas. Data were acquired between 50 and 1,200 *m/z*. The following parameters were then set: capillary voltages of 3,000 V, sampling cone voltages of 45 V, extraction cone of 4, source temperature of 100°C; desolvation temperature of 350°C and desolvation gas flow of 400 L/h. The chromatographic software MarkerLynx (Version 4.1, Waters, Milford, MA, USA) was used to process and obtain all the chromatographic data. The proposed identification of compounds was based on both the UV-Vis spectra (indicating the different classes of phenolic compounds), mass spectra (including molecular formulas of the parent ions, the related fragments and the lost moieties), comparison with pure standards when available, and with previous literature and reference data from the PubChem database (http://pubchem.ncbi.nlm.nih.gov). Molecular formulas were considered when the mass error was below 4 ppm.

#### Quantitative Analysis Using UPLC-DAD

For metabolites quantification, chromatographic analysis was carried out on a Shimadzu® LC-20AD (Japan) UPLC system equipped with a LC-20AD UPLC pump, a SIL-20AD auto sampler, a CTO-20AD thermostatted column compartment and an SPD-20AD photodiode array detector with a wavelength range of 190–700 nm. The analytical column was an ODS- Ultra Aqua C18 column (100 mm × 2.1 mm i.d., 3 μm particle size, Restek® (USA) protected with a Guard Cartridge (Restek®, Bellefonte, PA, USA). The column was maintained at 25°C. The mobile phase consisted of water solution of 0.1% trifluoroacetic (solvent A) and acetonitrile (solvent B). A linear gradient program at a flow rate of 0.200 was used: 0–5 min., 5% B; 5–25 min., 5–15% B; 25–35 min., 15–35% B; 35–45 min, 35–100% B; 45–48 min, 100–10% B; 48–50 min. Calibration curves were constructed for each standard using six concentrations with linearity range from 0 to 1,000 (*R*^2^ > 0.99). The concentrations of ferulic acid hexoside, procyanidin B2, eryodictyol-7-rutinoside and quercetin pentoside in samples were, respectively, calculated from the calibration curves constructed from pure ferulic acid, catechin, eryodictyol-7-rutinoside and quercetin standards (Supplementary Table 1). The assumption made for the quantification of ferulic acid hexoside was that its molar absorptivity is similar to that of its aglycone. The concentration of secondary metabolites in samples were expressed in μg/mg of dry weight.

### Quantification of Phenolic Compounds

#### Total Phenolic Contents (TPC)

Total phenolic content of the samples was estimated using Folin-Ciocalteu's reagent according to the modified procedure of Singleton and Rossi (35) with slight modifications. A 20 μL of the extract solution (prepared by re-dissolving 1 mg of extract in 1 mL of methanol) was mixed with 100 μL of Folin-Ciocalteu's reagent and incubated at room temperature (20°C) for 5 min. Following the addition of 300 μL of saturated aqueous sodium to the mixture, total phenolic contents were determined after 2 h of incubation in the dark at room temperature (20°C). The absorbance of the resulting blue solution was measured at 765 nm with a Jenway 7300 UV-vis spectrophotometer (United Kingdom). Quantification was done using the standard curve of gallic acid (Supplementary Table 1), and the results expressed as milligrams of gallic acid equivalent (GAE) per gram of dry weight.

#### Total Flavonoid Contents (TFC)

Analysis of total flavonoid content was carried out as described by Woisky and Salatino (34). A standard curve (*R*^2^ > 0.99) was constructed using six concentrations of quercetin (0, 25, 100, 500, 750, and 1,000 μg. mL^−1^) for use in extrapolating the concentration of flavonoids in samples. For this, 5 mg of quercetin was dissolved in 5 mL of 50% ethanol and then diluted to 12.5, 25, 50, 100, and 200 μg. mL^−1^. The standard solutions (2 mL) were separately mixed with 20 mL of 99% methanol, 1 mL of 5% aluminum chloride (wt/vol), 1 mL of 1 M potassium acetate, and the total volume was made up to 50 mL with distilled water. After incubation at room temperature (20°C) for 30 min, the absorbance of the reaction mixture was measured at 425 nm with a Jenway 7300 UV-vis spectrophotometer. An amount of 5% aluminum chloride was substituted by the same amount of distilled water in the blank. For the analysis of extracts from seeds of the different landraces, the procedure described above for the standard solutions were applied to 1 mL of each methanolic extracts (0.1 g/mL). The results were expressed as milligrams of quercetin equivalent (QUE) per gram of dry weight of plant material.

### Antioxidant Capacity (AC)

#### Free Radical-Scavenging Ability Using Stable DPPH Radical

DPPH free radical scavenging activity was determined according to the method described previously by Kim et al. (36). Briefly, 0.2 mM of methanolic DPPH solution was prepared and diluted with methanol until the absorbance reached 0.9 at 517 nm. One sixty microliter of the resulting solution was added to 40 μL sample extracts at different concentrations (1.95–250 μg/mL) in each of 96 well plates. After incubation in the dark at ambient temperature (20°C) for 30 min, the absorbance was measured at 517 nm using a micro-plate reader.

#### Free Radical-Scavenging Ability Using Stable ABTS Radical

ABTS free radical scavenging activity was evaluated by ABTS radical cation decoloration following the procedure described by Nenadis et al. (37). For this, 7 mM of ABTS ammonium was dissolved in water and treated with 2.45 mM of potassium persulfate, and the mixture was then allowed to stand at room temperature (20°) for 12–16 h to obtain a dark blue solution. This solution was diluted with methanol until the absorbance reached 0.7 at 734 nm. A 160 μL of the resulting solution was mixed with 40 μL methanolic extracts of the samples at different concentrations (1.95–250 μg/mL) in each well of 96 well plates. After incubation in the dark at ambient temperature (20°C) for 5 min, the absorbance was measured at 734 nm using micro plate reader.

For both the DPPH and ABTS assays, varying concentrations of Trolox and ascorbic acid were used as standards to construct the calibration curves. The % scavenging effect was calculated according to the following equation:

$$\begin{array}{l} \%\text{ scavenging}=\\ \left[1-\left(\text{Absorbance of sample}/\text{Absorbance of control}\right)\right]\times 100. \end{array}$$

Percent of scavenging effect was plotted against log concentration (μg/mL). The antioxidant activity of KG seed extracts was expressed as half-inhibitory concentration **(**IC_50_), which was defined as the concentration in μg/mL.

### Data Analysis

Statistical analyses were performed using Statistica version 10 software package. Data were analyzed using one-way ANOVA statistical model. Duncan's multiple range test (DMRT) was employed for mean comparisons among landraces. The difference was considered significant at *p* ≤ 0.05. All the experiments were performed in triplicates and data presented as mean ± standard error (SE).

Peak detection, alignment and the filtering of raw data were carried out using MarkerLynx v4.1. The parameters used included a retention time range of 1–18 min, a mass range of 100–1,000 Da, and a mass tolerance of 50 mDa. Noise elimination level was set at 1.00, the intensity threshold (counts) of collection parameters was set at 500; retention time tolerance was set at 0.4 min. The retention time and m/z data pair for each peak was determined by the software. Thereafter, the data was exported to PAST under the 3.06 environment which was used to construct various chemometric models and the relationships between the datasets determined. Hierarchical cluster analysis (HCA) and principal component analysis (PCA) were used to arrange unsupervised and supervised large data sets. PCA model was constructed to investigate clustering patterns and to obtain a general overview of the variance of KG metabolites among the landraces.

## Results

### Metabolites Identification Using UPLC-qTOF-MS Analysis

**Figure 1:**
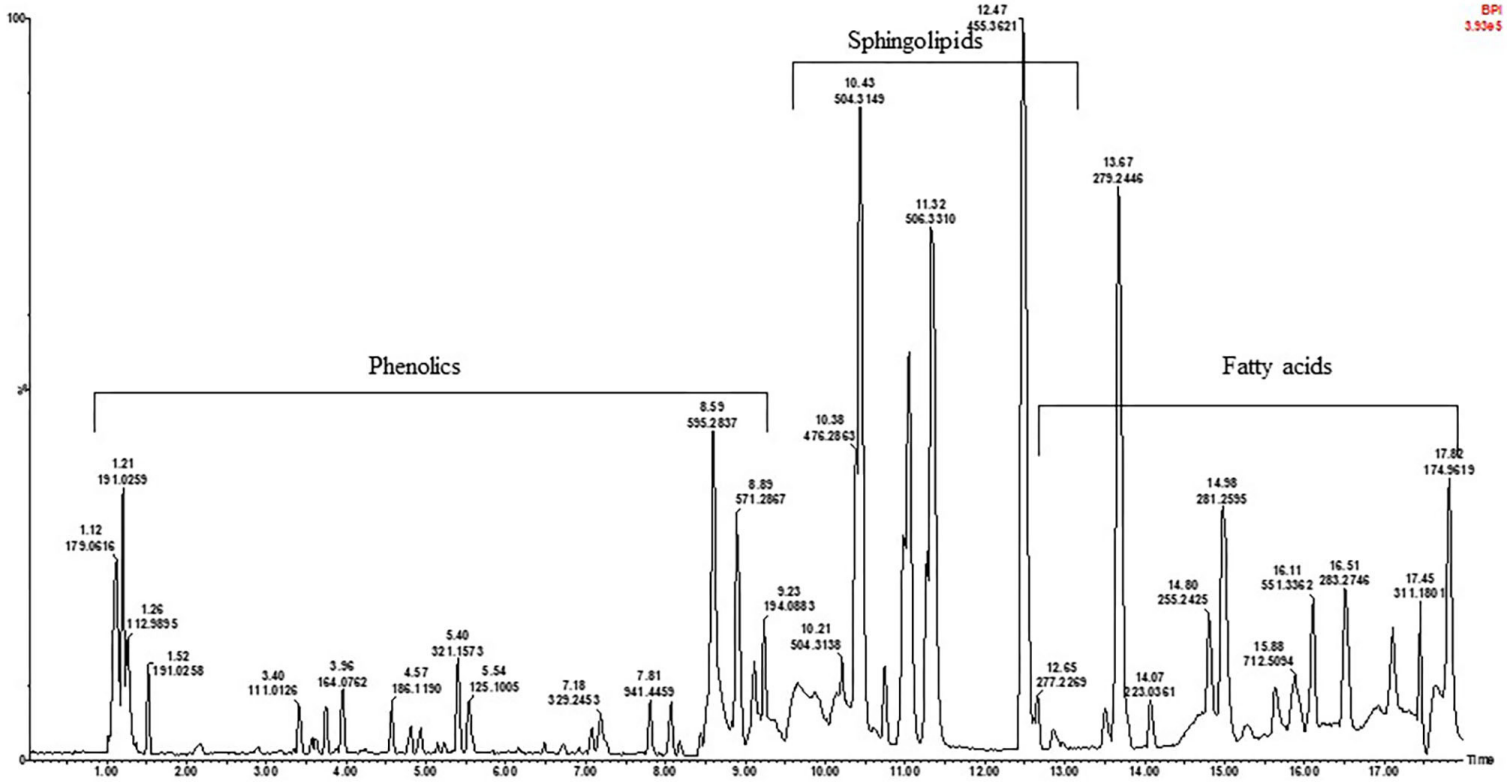
A representative UPLC-qTOF-MS fingerprint analysis of the black seeded Kersting's groundnut [Puffeun (KG1)] seed extract in negative ionization mode. Each peak represents individual compound identified, with details provided in Table 2.

To assess the metabolite composition of the different KG seeds, a non-targeted metabolites profile of the extracts was conducted *via* high resolution UPLC-qTOF-MS. As an example, the TIC (Total ion chromatogram) profile of the black seeded KG1 in the negative mode is presented in Figure 1. The chromatograms for the white and brown mottled landraces are shown in Supplementary Figure 2. A total of 57 compounds belonging to the sub-classes of phenolic acids, flavonoids, saponins, sphingolipids, and fatty acids were identified in the seeds tested (Table 2).

**Table 2:** Peak assignments of metabolites in acidified methanolic extracts of Kersting's groundnut (KG) seed analyzed using UPLC-qTOF-MS in negative ionization mode.

**Peaks**	**R_**t**_ (min)**	**Tentative assignment of identified compounds**	**UV (nm)**	**[M-H]^**−**^ (m/z)**	**Molecular formula**	**Error (mDA)**	**MS-MS fragmentation**
1	1.00	Gallic acid^a^	272	169.0142	C_7_H_5_${\text{O}}_{5}^{-}$	−2.2	125
2	1.13	Quercetin malonyl hexoside	256, 354	549.1440	C_24_H_21_${\text{O}}_{15}^{-}$	1.2	505, 300, 301
3	1.15	Ferulic acid hexoside	291, 329	355.1007	C_16_H_19_${\text{O}}_{9}^{-}$	−0.6	193, 175
4	1.16	Caffeic acid^a^	283, 320	179.0616	C_9_H_7_${\text{O}}_{4}^{-}$	0.8	135
5	1.17	*p*-coumaroylquininc acid	265, 317	471.0544	C_22_H_31_${\text{O}}_{11}^{-}$	−0.7	337, 191
6	1.19	Caffeic acid hexoside	287, 328	341.0934	C_15_H_17_${\text{O}}_{9}^{-}$	−0.6	193, 179
7	1.21	Quinic acid I^a^	nd	191.0111	C_7_H_11_${\text{O}}_{6}^{-}$	−2.0	nd
8	1.52	Quinic acid II	nd	191.0095	C_7_H_11_${\text{O}}_{6}^{-}$	−2.0	nd
9	3.20	Catechin hexoside	279	451.1066	C_21_H_23_${\text{O}}_{11}^{-}$	−0.6	162, 289
10	3.23	Catechin^a^	277	289.0598	C_15_H_13_${\text{O}}_{6}^{-}$	1.2	245, 179
11	3.28	Syringic acid	275, 328	197.0341	C_9_H_9_${\text{O}}_{5}^{-}$	0.9	182, 153, 135
12	3.43	*p*-coumaric acid^a^	229,312	163.0322	C_9_H_7_${\text{O}}_{3}^{-}$	0.8	119
13	3.64	Ferulic acid^a^	281, 323	193.0400	C_10_H_9_${\text{O}}_{4}^{-}$	0.9	178, 149
14	3.75	Sinapic acid^a^	238, 326	223.0495	C_11_H_11_${\text{O}}_{5}^{-}$	1.1	208, 179
15	3.80	Sinapic acid hexoside	279, 329	385.0896	C_17_H_21_${\text{O}}_{10}^{-}$	−0.8	233, 205
16	3.88	Epicatechin^a^	280	289.0597	C_15_H_13_${\text{O}}_{6}^{-}$	1.2	245, 179
17	3.98	Rutin^a^	254, 350	609.1842	C_27_H_29_${\text{O}}_{16}^{-}$	−0.9	343, 301, 300
18	4.09	Procyanidin dimer B1	232, 278	577.1085	C_30_H_25_${\text{O}}_{12}^{-}$	2.8	559, 425, 289
19	4.41	Procyanidin dimer B2	278	577.1074	C_30_H_25_${\text{O}}_{12}^{-}$	3.1	451, 425, 407, 289, 125
20	4.59	Procyanidin trimer B	279	865.1539	C_45_H_39_${\text{O}}_{18}^{-}$	2.9	713, 695, 577,289, 287
21	4.12	Naringin^a^	225, 280	579.1148	C_27_H_31_${\text{O}}_{14}^{-}$	2.9	459, 271
22	4.62	Gallocatechin Dimer	281	609.1154	C_30_H_25_${\text{O}}_{14}^{-}$	3.2	441, 423
23	4.81	Gallocatechin^a^	279	305.0630	C_15_H_13_${\text{O}}_{7}^{-}$	1.5	287, 179
24	4.84	Kaempherol-O-rutinoside	263, 340	593.1190	C_27_H_29_${\text{O}}_{15}^{-}$	0.7	285, 447
25	4.94	Quercetin dihexoside	255, 372	625.1478	C_27_H_29_${\text{O}}_{17}^{-}$	−0.6	300, 301
26	5.40	Digallic acid	272	321.1573	C_14_H_9_${\text{O}}_{9}^{-}$	0.9	169
27	5.54	Pyrogallic acid	270	125.1005	C_6_H_5_${\text{O}}_{3}^{-}$	0.9	nd
28	7.26	Vanillic acid hexoside^a^	254, 293	329.2181	C_14_H_17_${\text{O}}_{9}^{-}$	0.3	267
29	7.88	Dihydroxy-olean-enoic acid dihexosyl rhamnoside	nd	941.5104	C_48_H_77_${\text{O}}_{18}^{-}$	−5.4	915, 883, 795
30	8.43	Hydroxy oleanolic acid dirhamnosyl hexouronide	nd	939.4414	C_48_H_75_${\text{O}}_{18}^{-}$	0.6	913, 881
31	8.78	Dihydroxy-octadecadienoic acid	nd	311.2098	C_18_H_31_${\text{O}}_{4}^{-}$	5.5	275, 263
32	8.82	Apigenin dihexoside	267, 338	593.2672	C_27_H_29_${\text{O}}_{15}^{-}$	1.3	225, 269
33	9.33	Eryodictyol 7-O-rutinoside^a^	287	595.2837	C_27_H_31_${\text{O}}_{15}^{-}$	−1.1	287
34	9.64	*p-*coumaroyl hexoside	312	325.1961	C_15_H_17_${\text{O}}_{8}^{-}$	−0.7	163
35	10.21	LysoPE (20:2)	nd	504.3149	C_25_H_47_NO_7_P^−^	8.9	301
36	10.33	Quercetin pentoside	373	433.2163	C_20_H_17_${\text{O}}_{11}^{-}$	2.8	301
37	10.38	LysoPE (18:1)	nd	476.2863	C_23_H_43_NO_7_P^−^	7.8	279
38	10.42	LysoPC (18:1/2:0)	nd	564.3290	C_27_H_51_NO_9_P^−^	5.3	279
39	10.65	Trihydroxy-9,14-octadecatrienoic acid	nd	325.1973	C_18_H_29_${\text{O}}_{5}^{-}$	2.7	nd
40	10.95	LysoPE (16:0)	nd	452.2878	C_21_H_43_NO_7_P^−^	6.1	nd
41	11.05	LysoPE (18:0)	nd	480.3171	C_23_H_47_NO_7_P^−^	6.5	nd
42	11.28	LysoPE (18:2)	nd	478.3016	C_23_H_45_NO_7_P^−^	4.9	184
43	11.32	LysoPE (20:1)	nd	506.3310	C_25_H_49_NO_7_P^−^	7.1	281
44	11.35	LysoPC (18:0/2:0)	nd	566.3447	C_27_H_53_NO_9_P^−^	5.2	184
45	12.53	LysoPA (20:4)	nd	456.3652	C_21_H_47_NO_7_P^−^	7.4	nd
46	12.61	Oleanolic acid	nd	455.3304	C_30_H_47_${\text{O}}_{3}^{-}$	−0.5	nd
47	12.64	Linolenic acid	nd	277.2269	C_18_H_29_${\text{O}}_{2}^{-}$	−3.0	211
48	13.19	Hydroxy-palmitic acid	nd	271.2151	C_16_H_31_${\text{O}}_{3}^{-}$	1.9	180
49	13.50	Palmitoleic acid	nd	253.2257	C_16_H_29_${\text{O}}_{2}^{-}$	−3.2	113
50	13.88	Linoleic acid II	nd	279.2207	C_18_H_31_${\text{O}}_{2}^{-}$	−1.4	211
51	14.07	Linoleic acid I	nd	279.2439	C_18_H_31_${\text{O}}_{2}^{-}$	−1.7	211
52	14.80	Palmitic acid	nd	255.2203	C_16_H_31_${\text{O}}_{2}^{-}$	−2.5	113
53	14.98	Oleic acid I	nd	281.2747	C_18_H_33_${\text{O}}_{2}^{-}$	−1.6	183
54	15.48	Oleic acid II	nd	281.2598	C_18_H_33_${\text{O}}_{2}^{-}$	−1.6	183
55	15.62	Phenyl lactic acid	270	165.0454	C_9_H_9_${\text{O}}_{3}^{-}$	−2.3	nd
56	16.51	Stearic acid	nd	283.2746	C_18_H_35_${\text{O}}_{2}^{-}$	−1.7	nd
57	17.45	Dihydroxy octadecadienoic acid	nd	311.2211	C_18_H_31_${\text{O}}_{4}^{-}$	5.5	275, 263

*Rt, retention time; LPE, lysophosphatidylethanolamine; PLC, lysophosphatidylcholine; LPA, Lysophosphatidic acid. I, II, III stand for isomers*.

a *Confirmed using available standards; all the other compounds were identified based on MS/MS data; nd, not detected*.

The compounds detected from each chromatographic peak were tentatively characterized according to the corresponding spectral characteristics by comparison of their retention times and UV-vis spectra with those of available pure standards and confirmed by analysis of the mass spectra recorded for each peak. Other metabolites for which commercial standards were not available were identified based on their UV and MS spectra in comparison with previous reports on other grain legume sources of polyphenols.

### Identification of Hydroxybenzoic and Hydroxycinnamic Derivatives

In this study, 15 phenolic acid derivatives were successfully identified based on UPLC-qTOF-MS analysis and MS/MS fragmentation patterns. Of these phenolic acids, hydroxycinnamic acids, with maximum UV-Vis absorbance (λ_max_) within the range of 320 and 330 nm were the most represented. Peaks 3, 6, 15, 28, and 34 exhibited molecular ions [M-H]^−^ at *m/z* 355.1007, 341.0934, 385.0896, 329.2181, and *m/z* 325.1961 corresponding to the elemental formulas C_16_H_19_O_9_, C_15_H_17_O_9_, C_17_H_21_O_10_, C_14_H_11_O_9_, and C_15_H_17_O_8_, respectively (Figure 1; Table 2). The fragment ions at *m/z* 193 (C_10_H_10_O_4_), *m/z* 179 (C_9_H_8_O_4_), *m/z* 223 (C_11_H_12_O_5_), *m/z* 267 (C_8_H_8_O_4_), and *m/z* 163. (C_9_H_8_O_3_) were indicative of a loss of an hexosyl unit (162 amu; C_6_H_10_O_5_). Peaks 3, 6, 15, 28, and 34 were putatively identified as ferulic acid-hexoside, caffeic acid-hexoside, sinapic acid-hexoside, vanillic acid-hexoside and *p*-coumaroyl hexoside, respectively. Peak 26 presented a UV spectra similar to that of gallic acid but with different retention times. The MS presented the fragment [M-H]^−^ at *m/z* 321.1573 corresponding to 2 galloyl units. Based on the MS data and previous literature (38), this compound was assigned to the hydrolysable tannin, digallic acid. The above analytical approach also led to the proposed identification of peaks 27 and 55 as pyrogallic acid and phenyl lactic acid, respectively.

### Identification of Flavonoids

Compounds 9, 10, 16, and 18–20 showed UV spectra with shape and maximum wavelength which were similar to that of catechin. Peak 20 had molecular ion [M–H]^−^ with *m/z* 865.1539 and λ_max_ of 279 nm and was identified as procyanidin trimer with B-type linkage. Compounds 22 and 23 presented molecular ions [M-H]^−^ at *m/z* 609.1154 and 305.0630, respectively, and were tentatively identified as gallocatechin dimer and gallocatechin, even if the fragment ions from their MS/MS fragmentations could not be obtained. The UV spectra of compounds 2, 25 and 36 were similar to that of quercetin. The MS (ESI) analysis presented their peaks at m/z 549.1440, 625.1478, and 433.2163, respectively. The fragmentation pattern analysis of peaks 25 and 36 revealed a loss of two hexose units and pentose, respectively. The resulting fragments at *m/z* 301 (C_15_H_10_O_7_) for both peaks could be attributed to quercetin, suggesting that in these compounds, quercetin is linked to two hexose units and one pentose, respectively. Peak 2 (λ_max_ = 354 nm) had a molecular ion [M-H]^−^ at *m/z* 549.1440 and showed MS/MS fragment at *m/z* 301.0348, corresponding to a loss of 248 amu. This indicated a loss of 162 amu for hexose plus 86 amu for malonic acid. Apigenin dihexoside is proposed for peak 32 with molecular ion [M-H]^−^ at *m/z* 593.2479. In the MS/MS spectra, the loss of two hexose (322 amu) moieties gave a fragment ion at *m/z* 270, which corresponds to the structure of apigenin. Following the above analytical approach, peaks 17, 21, 24, and 33 were proposedly identified as rutin, naringin, kaempherol 7-rutinoside, and eriodictyol 7-rutinoside, respectively.

### Identification of Fatty Acids and Sphingolipids

In the second half of the chromatogram, peaks corresponding to fatty acids (saturated and unsaturated) and sphingolipids were identified. Several signals of unsaturated fatty acids were assigned as oleic acid (53), linoleic acid (50), linolenic acid (47), and palmitoleic acid (49). This was evident from the high-resolution masses at *m/z* 281.2747, 279.2439, 277.2269, and 253.2257 corresponding to the molecular formula C_18_H_33_${\text{O}}_{2}^{-}$, C_18_H_31_${\text{O}}_{2}^{-}$, C_18_H_29_${\text{O}}_{2}^{-}$, and C_16_H_29_${\text{O}}_{2}^{-}$ (39, 40). Other signals were attributed to saturated fatty acids, i.e., stearic acid (56) and palmitic acid (52) as evident from the high-resolution mass at *m/z* 283.2746 and 255.2203; with predicted molecular formulae of C_18_H_35_${\text{O}}_{2}^{-}$ and C_16_H_31_${\text{O}}_{2}^{-}$, respectively.

In this same half of the chromatogram, several intense peaks which presented an even molecular mass of 452.2878, 456.3652, 476.2863, 478.3016, 480.3171, 504.3149, 506.3310, 564.3290, and 566.3447 with predicted molecular formula of C_21_H_43_NO_7_P^−^, C_21_H_47_NO_7_P^−^, C_23_H_43_NO_7_P^−^, C_23_H_45_NO_7_P^−^, C_23_H_47_NO_7_P^−^, C_25_H_47_NO_7_P, C_25_H_49_NO_7_P^−^, C_27_H_51_NO_9_P^−^, and C_27_H_53_NO_9_P^−^, respectively, were observed. The difference of 2 amu between these masses is indicative of the presence of unsaturation (41).

### Identification of Saponins

The seeds of the KG landraces contained 3 triterpenoid saponins of oleanane series which were detected in peaks 29, 30, and 46, containing 3-hydroxy-12-olean-28-oic acid (*m/z* 455) aglycones based on the report by Frang et al. (41).

### Total Phenolic and Flavonoid Contents, and Antioxidant Activity of KG Seed Extracts

The ability of the methanolic extracts prepared from KG seeds to scavenge free radicals using DPPH and ABTS assays in comparison to trolox and ascorbic acid used as positive controls were evaluated (Table 3), with low IC_50_ values indicating a strong capacity to quench free radicals and *vice versa*. With respect to DPPH assay, the tested landraces recorded significantly (*p* < 0.05) higher IC_50_ values compared to trolox and ascorbic acid used as positive controls. The IC_50_ values ranged from 5.12 for KG3 to 95.21 μg/mL for KG8 (Table 3). The ABTS results revealed marked variations in the IC_50_ values of landraces, with values ranging between 11.77 for KG3 to 63.65 μg/mL for KG8. The landrace KG3 recorded similar IC_50_ value as the trolox control, and together with the remaining landraces had lower values when compared to the ascorbic acid control (Table 3). Among the landraces, KG3 appears to exhibit the most antioxidant activity as evidenced by its lower IC_50_ values in both assays. Conversely, the markedly higher IC_50_ values of KG8 with respect to DPPH and ABTS assays indicates least antioxidant activity of the landrace compared to the others (Table 3).

**Table 3:** Total phenolic, flavonoid and anthocyanin contents as well as the antioxidant capacity of Kersting's groundnut (KG) seeds.

**Landraces**	**TPC^a^**	**TFC^b^**	**DPPH**	**ABTS**
	**(mg GAE/g)**	**(mg QUE/g)**	**IC_**50**_ (μg/mL)**	**IC_**50**_ (μg/mL)**
KG1	9.44 ± 0.03a	3.01 ± 0.3a	9.17 ± 0.12bc	18.44 ± 0.30ef
KG2	1.73 ± 0.05h	0.54 ± 0.03c	11.17 ± 0.10b	23.08 ± 0.29d
KG3	6.45 ± 0.16c	2.78 ± 0.30a	5.12 ± 0.10d	11.77 ± 0.14g
KG4	5.16 ± 0.01e	2.49 ± 0.18ab	7.70 ± 1.35cd	19.91 ± 0.55e
KG5	7.96 ± 0.15b	2.82 ± 0.15a	9.10 ± 0.92bc	29.85 ± 1.69b
KG6	5.57 ± 0.19d	2.11 ± 0.23b	6.57 ± 1.44cd	16.83 ± 0.52f
KG7	3.61 ± 0.07g	2.75 ± 0.11a	6.62 ± 0.62cd	26.91 ± 1.90c
KG8	4.07 ± 0.02f	2.89 ± 0.08a	95.21 ± 1.62a	63.65 ± 0.43a
Trolox	–	–	2.38 ± 0.08e	10.91 ± 0.39g
Ascorbic acid	–	–	2.15 ± 0.40e	3.43 ± 0.78h
F statistics	516.8^***^	16.8^***^	996.0^***^	330.1^***^

a *Total phenolic content is expressed as milligrams of gallic equivalent (GAE) concentration per gram of dry seeds*.

b Total flavonoid content is expressed as milligrams of quercetin equivalent (QUE) concentration per gram of dry seeds. Values (means ± SE) with dissimilar letters in a column are significantly different at

*** *p < 0.001*.

Furthermore, TPC and TFC were determined using colorimetric methods, and results were expressed as mg of gallic acid and quercetin equivalents, respectively, per gram of seed dry weight. The total phenolic content showed significant (*p* < 0.05) variation among the landraces and ranged from 1.73 to 9.44 mg GAE/g^1^ of dry weight (Table 3). The highest and lowest TPC values were observed in the black seeded KG1 (9.44 mg GAE/g) and white seeded KG2 (1.73 mg GAE/g), respectively (Table 3). Total phenolic content decreased in the order KG1 > KG5 > KG3 > KG6 > KG4 > KG8 > KG7 > KG2. Clearly, the KG seeds in this study exhibited marked variations in flavonoid contents (Table 3). The white seeded KG2 recorded the least total flavonoid content (0.53 mg QUE/g) compared to the remaining landraces which had higher but similar total flavonoid contents, ranging from 2.11 to 3.01 mg QUE/g of dry weight (Table 3). Whereas, the white pigmented KG2 showed the least total flavonoid content, the variation in total flavonoid content was not significant (*p* > 0.05) among the remaining dark-seeded landraces in this study.

To elucidate the relationship among the different KG landraces, the 1/IC_50_ values were correlated to the TPC, TFC, ferulic acid hexoside, procyanidin B2, eryodictyiol-7-rutinoside and quercetin pentoside contents of the eight samples using Pearson's correlation analysis, and several meaningful correlations were observed (Supplementary Table 2). Firstly, the ABTS correlated with DPPH, TPC, TFC, and all individual phenolics, except for quercetin pentoside. A significant correlation between TPC and antioxidant capacity (ABTS and DPPH) was also observed (r = 0.79, *p* < 0.05 and 0.67, *p* < 0.05, respectively) while a similar trend was also noticed between TFC and antioxidant activity based on ABTS (r = 0.87, *p* < 0.01) and DPPH (r = 0.83, *p* < 0.05) assays (Supplementary Table 2).

### Multivariate HCA and PCA Analysis of UPLC-qTOF-MS Data

**Figure 2:**
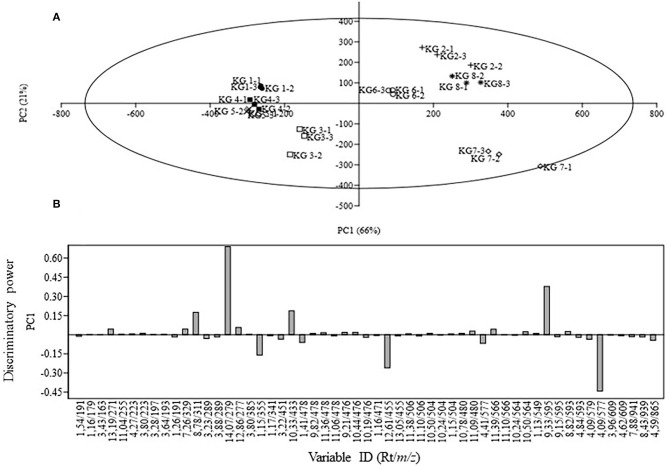
Principal component analysis of eight Kersting's groundnut (KG) landraces analyzed using UPLC-qTOF-MS. **(A)** Score plot of PC1 vs. PC2 scores; **(B)** Loadings plot for PC1.

Due to the complexity of the data acquired, as reflected on the chromatograms, chemometric analysis of the biochemical profiles derived from the eight KG landraces was used to evaluate the contribution of the major phenolic compounds to their diversity and relationships. Differences in the chemical composition and concentrations of individual compounds were evident from the UPLC profiles of the tested landraces with varying susceptibility. The extracts from the samples were analyzed in both positive and negative ionization modes, and the negative mode appeared to be more sensitive. From the 24 samples, 5,232 and 4,680 mass signals were extracted by MarkerLynx software from the UPLC-qTOF-MS data set acquired in negative and positive modes, respectively. Since the PCA results obtained from both positive and negative modes were similar, the results derived from the negative mode are presented herein. Triplicate measurements from the same sample were found to be reproducible, as the scores of replicate measurements were close and/or superimposed (Figure 2A). Considering the 57 variables as analytical data, the PCA was able to discriminate among landraces. Five principal components (PCs) were required to capture 99% of the total variance. The main PC that differentiated the KG samples was PC1, which accounted for 66% of the variance, while PC2 explained 21% of the variation captured. Landraces KG2, KG6, KG7, and KG8 were positioned on the right side (positive PC1 values) whereas landraces KG1, KG3, KG4, and KG5 were located on the left side of the vertical line (representing negative PC1 values) with KG7 being the most distant from the others. The PC1/PC2 scores plot (Figure 2A) revealed the existence of three distinct clusters distributed over three regions. The segregation observed in the PCA score plot can be explained in terms of the identified compounds using the loadings plot that revealed the compounds having significant effect on the principal component (PC1). Examination of the loadings plot (Figure 2B) showed that the variables refer to signals with retention/mass ratio (r_t_/*m/z*) of 1.15/355, 4.09/577, 9.33/595, 10.33/433, 12.61/455, and 14.07/279, respectively corresponding to procyanidin B2, eriodictyol-7-O-rutinoside, oleanolic acid, quercetin pentoside, ferulic acid hexoside, linoleic acid, and dihydroxy-octadecadienoic acid, and were the most involved in discriminating among the KG landraces. Dihydroxy-octadecadienoic acid, quercetin pentoside, and eriodictyol-7-O-rutinoside contributed positively to PC1 while ferulic acid hexoside, oleanic acid, and procyanidin B2 contributed negatively.

**Figure 3:**
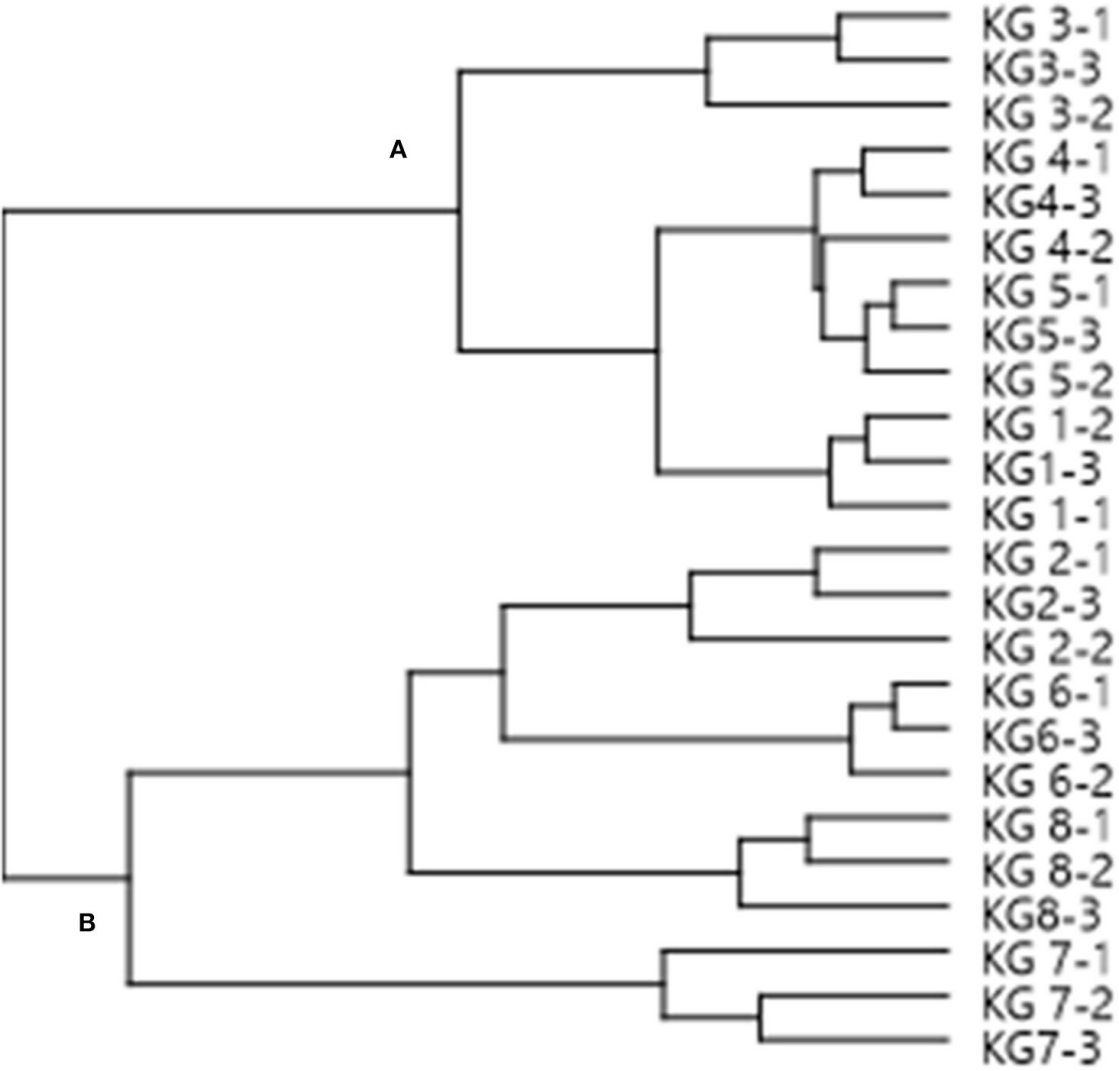
A dendrogram of Kersting's groundnut (KG) landraces based on group cluster analysis, using MS chemical profiles as analytical data. The MS chemical profiles of three replicate samples per landrace were used for the analysis. The name of each landrace is followed by a hyphen (-), and then the replicate number 1, 2 or 3.

HCA was also used as an additional tool to explain the segregation between the different landraces. From the dendrogram (Figure 3), two distinct clusters with 4 landraces were observed. Examination of Cluster **A** showed that KG3 is more related to KG4 toward KG5 and KG1. In Cluster **B**, KG7 was the most distant landrace in comparison to KG2, KG6, and KG8, while all the other landraces grouped in one separate sub-cluster. The clustering of landraces mirrored the patterns of their total phenolic contents. For example, the samples in Cluster **A** had greater phenolic contents and antioxidant properties compared to those in Cluster **B**.

Metabolites showing differential accumulation among the seeds were subjected to absolute quantification using pure standards (Table 4). In agreement with the PCA and HCA results, the highest concentrations of ferulic acid hexoside, procyanidin B2 were recorded in landraces KG1, KG3, KG4, and KG5. As shown in Table 4, ferulic acid hexoside was the most predominant compound in the KG seeds, with the highest concentration detected in KG1 with a mean value of 441.31 ± 55.80 μg/g DW, while KG7 recorded a much lower concentration (51.78 ± 11.39 μg/g DW). Procyanidin was found in traces in the white seeded KG2 while eriodictyol-7-*O*-rutinoside was not detected in KG7.

**Table 4:** Quantitative analyses of metabolites showing differential accumulation in seeds of Kersting's groundnut (KG) landraces.

**Peak no**.	**Name**	**mol. ion [m/z]**	**KG1**	**KG2**	**KG3**	**KG4**	**KG5**	**KG6**	**KG7**	**KG8**	**μg/mg**
3	Ferulic acid hexoside	355	441.31	138.57	287.85	354.30	287.78	229.36	51.78	155.59	Average
			55.80	11.87	26.13	5.57	32.87	12.93	11.39	18.59	Std dev
19	Procyanidin B2	577	11.24.	trace	18.25	16.67	18.12	8.39	11.92	1.86	Average
			0.27	nd	3.69	0.11	0.48	0.11	0.26	0.32	Std dev
33	Eryodictyol-7-rutinoside	595	7.49	10.22	6.83	7.58	5.97	13.95	3.26	nd	Average
			0.36	2.60	0.02	0.76	1.34	0.28	0.25	nd	Std dev
36	Quercetin pentoside	433	7.94	11.55	11.80	6.06	5.44	15.20	63.85	14.14	Average
			0.86	1.97	0.33	0,27	0.30	0,18	10.45	2.57	Std dev

*Trace, detected in trace amount; nd, not detected*.

## Discussion

### Phenolic Acids Composition of Kersting's Groundnut (KG) Seeds

Although KG seeds have been reported to have medicinal value, not much information exists on the crop's metabolite composition. To explore the potential of KG as a source of nutraceuticals, this study screened for the presence and relative concentrations of metabolites in the seeds of different landraces of this orphan legume. The findings revealed the presence of diverse classes of secondary metabolites in the seeds tested, some of which exhibited variable abundance among the landraces. For example, several phenolic acid derivatives such as ferulic acid-hexoside, caffeic acid-hexoside, sinapic acid-hexoside, vanillic acid-hexoside, and *p*-coumaroyl hexoside were detected in the seeds tested. A previous study by Nyau et al. (21) identified similar compounds in seeds of Bambara groundnuts, another underutilized legume indigenous to Africa and known for its nutritional values. In this study, the identity of other hydroxycinnamic and benzoic acids was deduced from the fragmentation patterns of their respective mass spectra, which corresponded to gallic acid, caffeic acid, *p*-coumaroylquinic acid, quinic acid, syringic acid, *p*-coumaric acid, ferulic acid, and sinapic acid. These compounds are ubiquitous in the seeds of many grain legumes such as cowpea, pea, common bean and horse gram (42–45). Even though the UV spectra of peak 26 was similar to that of gallic acid, the retention times were different; nevertheless, the compound was identified as the hydrolysable tannin, digallic acid, based on the MS data previously reported by El Sissi et al. (38). The presence of these phenolic acids in plants is linked to diverse functions, including nutrient uptake, protein synthesis, enzymatic, and photosynthetic activities (46). For example, gallic acid identified in seeds of the test KG landraces is reported to act as an antioxidant and allochemical, and is also known for its anticancer, antiviral and astringent properties (46). Quinic acid on the other hand, is involved in DNA repair, a reduction in the influence of lifestyle factors on the risk to disease as well as the inhibition of cell proliferation (47). The observed phenolic profile of the tested KG seeds highlights the potential health-promoting benefits of the crop when included in diets.

### Flavonoids in Seeds of the Orphan Kersting's Groundnut

Along with the phenolic acids, several classes of flavonoids including catechin and procyanidin B dimers were detected in seeds of the KG landraces tested. For example, compounds 18 and 19 had a similar UV spectrum as that of procyanidin oligomers, and had molecular ions [M-H]^−^ at *m/z* 577.1085 and 577.1074 corresponding to procyanidin type B dimers (48). Since procyanidins B are mostly catechin dimers, these compounds, previously identified in the seed and seed coats of lentil, pardina lentil, pea and common bean were assigned as procyanidin B1 and procyanidin B2 because of the MS^2^ fragment ion [M-H]^−^ at *m/z* 289 which is characteristic of catechin monomer (49). The presence of procyanidin type B dimers has been described in seeds of most grain legumes, and their synthesis may depend on the legume's phenotype (50, 51). Moreover, B-type procyanidin trimers which were found in the test landraces were earlier detected in the seeds of cowpea, adzuki bean, pea and lentil (4, 49). Of the flavonoids detected in this study, gallocatechin is reported to improve lipid metabolism and contributes to the prevention of metabolic syndrome, and was earlier reported in seeds of pea and lentil (51, 52). The presence of quercetin dihexoside and quercetin pentoside in the test KG landraces was previously reported in the seeds of cowpea (53). In diabetic rats, quercetin was found to exert antidiabetic properties by increasing the regeneration of pancreatic islets and the release of insulin (54). Acyl groups such as acetyl or malonyl usually occur as 6″-*O*-acetylglucoside or 6″-*O*-malonylglucoside (43). Thus, peak 2 was tentatively identified as quercetin-3-(6″-malonoyl)-glucoside. Flavonoids containing 6″-malonoyl-glucoside groups have been reported in the seeds of cowpea and soybean (36, 53). This study further identified compounds such as apigenin (peak 32), rutin (17), naringin (21), kaempherol 7-rutinoside (24), and eriodictyol 7-rutinoside (33) which were previously reported in seeds of pardina lentil (55). In addition to the diverse roles of these classes of flavonoids in the signaling processes during the legume-rhizobium symbiosis (14), they are also linked to reduced incidence of cancers and cardiovascular diseases in humans (56). Aside their antioxidant activities, flavonoids have also been implicated in the regulation of metabolic functions of the gut microbiota in favor of human health (57).

### Fatty Acids and Sphingolipids Detected in the Seeds of Kersting's Groundnut

From the chromatogram in this study (Figure 1), peaks corresponding to unsaturated fatty acids (e.g. oleic, linoleic, linolenic, and palmitoleic acids) and saturated acids (e.g., stearic acid and palmitic acid) were identified in the seeds tested. Fatty acids are metabolites that take part in complex metabolic pathways and play major biological roles in organisms. Whereas, saturated fatty acids are associated with adverse health effects, unsaturated fatty acids are thought to be protective (19). The chromatogram also presented several other intense peaks corresponding to sphingolipid conjugates which are known for their roles in a wide range of biological processes and functions in human systems, including the management of several diseases such as cancer, obesity and atherosclerosis (20).

### Antioxidant Activities and Relative Concentrations of Total Phenolic Compounds in Seeds of Kersting's Groundnut

Legumes are natural sources of antioxidants that can be used to manage neurodegenerative diseases. In the management of these diseases, scavenging reactive oxygenated species is an important mechanism of antioxidant action (58). The methanolic extracts from seeds of the test KGs exhibited significant variations in antioxidant activities, with the brown mottle seeded KG3 showing the highest activity than the other landraces based on DPPH and ABTS assays. For example, the DPPH assay revealed markedly higher (*p* < 0.05) IC_50_ values in the landraces when compared to trolox and ascorbic acid used as positive controls, with values ranging from 5.12 μg/mL for the extract from KG3 to 95.21 μg/mL for the extract from KG8. The landrace KG8 was found to show the least antioxidant activity based on its greater IC_50_ values in both DPPH and ABTS assays (Table 3).

In this study, the total phenolic content in seeds decreased in the order KG1 > KG5 > KG3 . KG6 > KG4 > KG8 > KG7 > KG2, a finding consistent with earlier reports which showed that legumes with light seed coat colors tend to have lower phenolic content when compared to those with dark-pigmented seed coats (59, 60). Consequently, the total flavonoid content also ranged from a low 0.53 mg QUE/g in the white seeded KG2 to a high 2.11–3.01 mg QUE/g in the brown and black seeded landraces. Ojwang et al. (53) also reported greater accumulation of flavonols in red seeded cowpea when compared to black, green and white seed coat phenotypes. The total flavonoid contents observed in seeds of the test KG landraces were within the range reported for cowpea (61).

The antioxidant activity of the KG seeds studied can partly be attributed to their phenolic composition, especially the ability of polyphenolic compounds to quench free radicals. The positive correlation between total phenolic content, total flavonoid content and the individual phenolic compounds with antioxidant capacity indicated that phenols and flavonoids in KG seeds improved its antioxidant properties. These findings confirm that seed polyphenolic content can be considered as a predictor of the antioxidant activity (*in vitro*) as indicated by earlier studies on grain legumes (4, 62). Nevertheless, in evaluating the contribution of polyphenolics to antioxidant activity, Cardador-Martinez et al. (63) found that 40–71% of the free scavenging is linked to total phenolic content, and that flavonoids were responsible for only 20–30% of the antioxidant activity. Despite the relatively lower total phenolic and flavonoid contents of the white seeded KG2 in this study, the extracts of the landrace were among those with higher antioxidant properties based on ABTS assays. Thus, although total phenolic and flavonoid contents were directly correlated with potential antioxidant activities in this study, the few discrepancies may be due to the reported contribution of non-phenolic compounds to the total antioxidant activities of seed extracts (64, 65). Considering the marked differences observed in the phytochemical profiles and antioxidant activities of the seeds tested, and although not addressed here, such observations can be attributed to the impact of edaphoclimatic conditions, such as light exposure, temperature, and soil properties, which have been increasingly recognized as having a direct impact on both chemical composition and biological activity (66). Moreover, Cheng et al. (67) also reported a marked effect of plant processing and extraction methods on the phytochemical and antioxidant properties of grape residues. However, the effect of these factors on the phytochemical composition and antioxidant properties of KG are yet to be determined.

A PCA analysis of the metabolites identified revealed marked differences in the eight KG landraces, with KG7 being the most distant landrace due to its relatively unique metabolite composition. The PCA grouped the test landraces into three major clusters, with procyanidin B2, eriodictyol-7-*O*-rutinoside, oleanolic acid, quercetin pentoside, ferulic acid hexoside, linoleic acid, and dihydroxy-octadecadienoic acid showing a greater contribution to the discrimination among the landraces. However, the HCA analysis grouped the landraces in two major clusters which mirrored the patterns in their phenolic contents, with samples in Cluster **A** exhibiting greater phenolic contents than their counterparts in Cluster **B** (Figure 3).

## Conclusion

By means of UPLC-qTOF-MS and UPLC-DAD, the chemical profiles of eight KG landraces were deciphered. A total of 57 secondary metabolites were putatively identified and some of them quantified. The chemical fingerprints of all the analyzed landraces were dominated by phenolics, sphingolipids and fatty acids. The results from this study provide a useful documentation on the chemical profile of KG seeds using untargeted large-scale metabolite analysis. The variability in phenolic content among the different landraces could provide an interesting and valuable source of information for breeding programs to improve the cultivation and utilization of KGs. Our findings therefore highlight the potential of this under-utilized crop as a source of health-promoting foods due to the presence of diverse classes of nutraceutical compounds. These results have implications for the role of compounds identified in the dietary intake by malnourished African populations, and also highlight the potential of KG as an alternative subsistence crop that can be used to combat malnutrition in sub-Saharan Africa. The compounds detected have been variously reported for their biological functions and can be exploited for improved human health and nutrition subject to more research that should include cell-based assays of seed extracts or it's other formulations.

## Data Availability Statement

The original contributions presented in the study are included in the article/Supplementary Materials. Further inquiries can be directed to the corresponding authors.

## Author Contributions

ATT designed and performed the experiments, interpreted the results, and drafted the manuscript. MM collected the plant materials, analyzed the data, interpreted the results, and took part in drafting the manuscript. FDD provided the funding, conceived the idea, supervised the work, edited, and approved the final version of the paper. All authors contributed to the article and approved the submitted version.

## Conflict of Interest

The authors declare that the research was conducted in the absence of any commercial or financial relationships that could be construed as a potential conflict of interest.
