# Supplementary material for: Metabolite Fingerprinting of Kersting's Groundnut [Macrotyloma geocarpum (Harms) Maréchal & Baudet] Seeds Using UPLC-qTOF-MS Reveals the Nutraceutical and Antioxidant Potentials of the Orphan Legume
Source: Front Nutr. 2020 Dec 15;7:593436. doi: 10.3389/fnut.2020.593436 (PMC7770220; doi:10.3389/fnut.2020.593436)
Supplement: Supplementary file 1 [file Table_1.docx]

**ELECTRONIC SUPPLEMENTARY INFORMATION**

Metabolite Fingerprinting of Kersting’s Groundnut [*Macrotyloma geocarpum* (Harms) Marechal & Baudet] Seeds using UPLC-qTOF-MS reveals the Nutraceutical and Antioxidant Potentials of the Orphan Legume.

**Supplementary Figure 1.** Pictures of Kersting’s groundnut seeds used for this study.


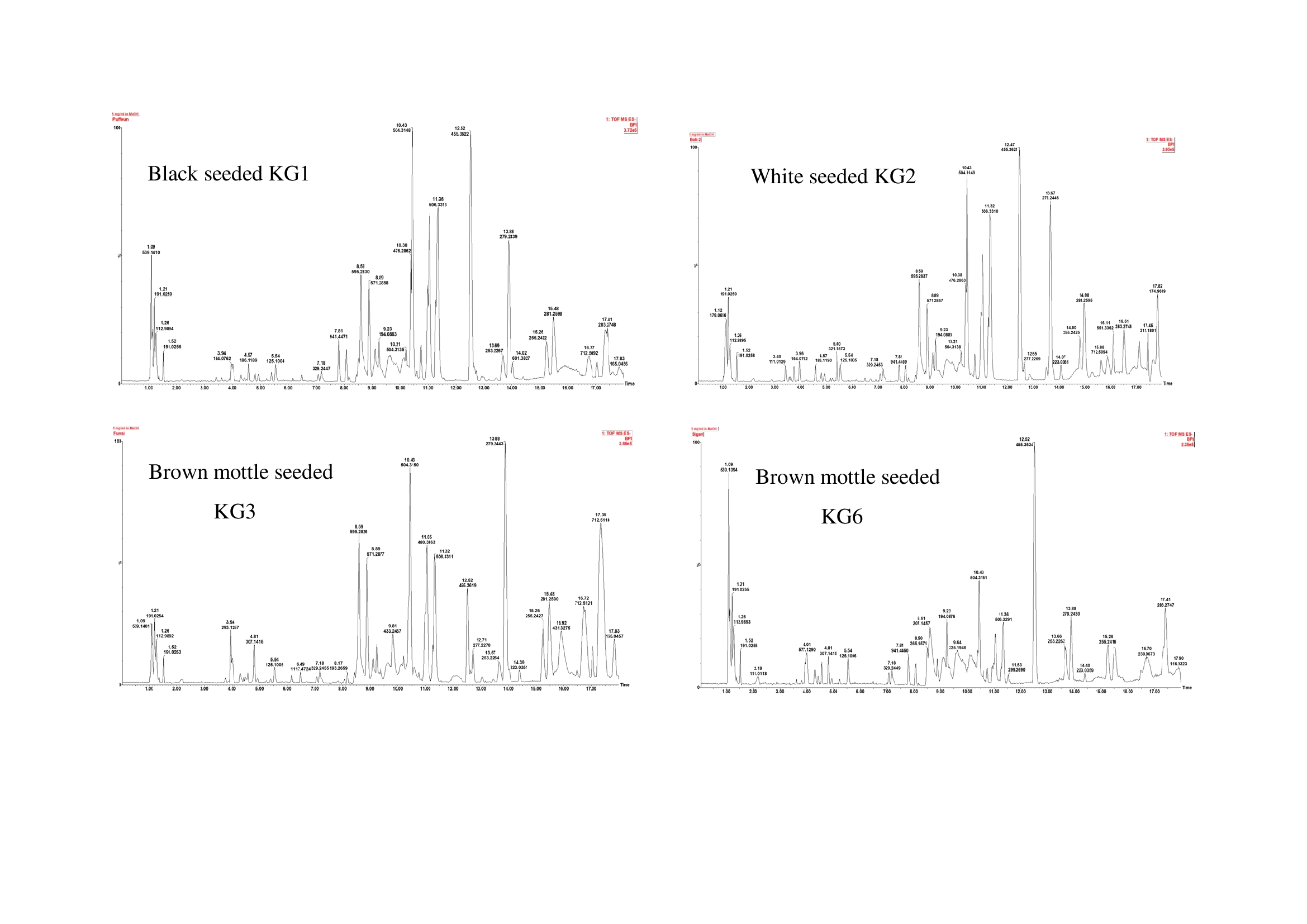


**Supplementary Figure 2: UPLC chromatograms of black, white and brown mottle seed coated Kersting’s groundnuts seeds.**

**Supplementary Table 1. Equations of the calibration curves from pure standards used for the quantification of metabolites**

| Pure standards | Linear range | Regression equation | R^2^ | LOD | LOQ |
| --- | --- | --- | --- | --- | --- |
|  | (µg/mL) |  | - | (µg/mL) | (µg/mL) |
| Gallic acid | 0-1000 | $\text{y}\text{ }\text{=}\text{ }\text{1.09}\text{x}\text{-0.024}$ | 0.9908 | 3.5970 | 17.9850 |
| Quercetin | 0-1000 | $\text{y}\text{ = 4.89×}\text{10}^{\text{-2}}\text{x}\text{ + 0.420}$ | 0.9977 | 0.1616 | 0.8080 |
| Ferulic acid | 0-1000 | $\text{y}\text{ }\text{=}\text{ }\text{1.07×}\text{10}^{\text{-5}}\text{x}-\text{0.072}$ | 0.9953 | 5.54 x 10^-5^ | 2.77 x 10^-4^ |
| Catechin | 0-1000 | $\text{y}\text{ }\text{=}\text{ }\text{1.38×}\text{10}^{\text{-4}}\text{x}-\text{0.061}$ | 0.9960 | 4.56 x 10^-4^ | 2.28 x 10^-3^ |
| Eryodictyol | 0-1000 | $\text{y}\text{ }\text{=}\text{ }\text{2.81×}\text{10}^{\text{-5}}\text{x}-\text{0.055}$ | 0.9982 | 4.07 x 10^-4^ | 2.03 x 10^-3^ |

*y* = concentration, *x*= peak area or absorbance

**Supplementary Table 2. Pearson’s correlation coefficients between antioxidant activity and total phytochemical contents**

| Variable | 1 | 2 | 3 | 4 | 5 | 6 | 7 |
| --- | --- | --- | --- | --- | --- | --- | --- |
| 1.ABTS (1/IC_50_) | 1 |  |  |  |  |  |  |
| 2.DPPH(1/IC_50_) | 0.96^***^ | 1 |  |  |  |  |  |
| 3.Total phenolic content | 0.79^*^ | 0.67^*^ | 1 |  |  |  |  |
| 4.Total flavonoid content | 0.87^**^ | 0.83^*^ | 0.67^ns^ | 1 |  |  |  |
| 5.Ferulic acid hexoside | 0.40* | 0.15^ns^ | 0.80*** | 0.34ns | 1 |  |  |
| 6. Procyanidin B2 | 0.48* | 0.59** | 0.61** | 0.50* | 0.47* | 1 |  |
| 7. Eriodyctyiol-7-rutinoside | 0.54** | 0.44* | 0.06^ns^ | -0.48* | 0.25^ns^ | 0.01^ns^ | 1 |
| 8. Quercetin pentoside | -0.19^ns^ | 0.16^ns^ | -0.38^ns^ | 0.12^ns^ | -0.68*** | -0.06^ns^ | -0.06^ns^ |

Pearson’s correlation analysis was conducted using averaged values of each variable (n=8)

^***, **, *^ and ^ns^ indicate significance at *p*<0.001, 0.01, 0.05 and non-significance, respectively.
